# Research progress on the regulation of production traits by gastrointestinal microbiota in dairy cows

**DOI:** 10.3389/fvets.2023.1206346

**Published:** 2023-08-01

**Authors:** Lily Liu, Peifu Wu, Aiwei Guo, Yajin Yang, Fenfen Chen, Qin Zhang

**Affiliations:** ^1^College of Life Science, Southwest Forestry University, Kunming, China; ^2^College of Animal Science and Technology, China Agricultural University, Beijing, China; ^3^College of Animal Science and Technology, Shandong Agricultural University, Tai’an, China

**Keywords:** dairy cows, production traits, health, rumen microbiota, gut microbiota

## Abstract

The composition and abundance of microorganisms in the gastrointestinal tract of cows are complex and extensive, and they play a crucial role in regulating nutrient digestion, absorption, maintaining digestive tract stability, and promoting the production and health of the host. The fermentation carried out by these microorganisms in the gastrointestinal tract is fundamental to the health and productivity of cows. Rumen microorganisms produce the majority of enzymes required to break down feed substrates, such as cellulose, protein, lipids, and other plant materials, through fermentation. This process provides energy metabolism substrates that satisfy approximately 70% of the host’s energy requirements for physiological activities. Gut microorganisms primarily decompose cellulose that is difficult to digest in the rumen, thereby providing heat and energy to the hosts. Additionally, they have an impact on host health and productivity through their role in immune function. Understanding the composition and function of the cow gut microbiota can help regulate dairy cattle breeding traits and improve their health status. As a result, it has become a popular research topic in dairy cattle breeding. This article provides a review of the composition, structure, physiological characteristics, and physiological effects of the cow gut microbiota, serving as a theoretical foundation for future studies that aim to utilize the gut microbiota for dairy cattle breeding or improving production traits. It may also serve as a reference for research on gut microbiota of other ruminants.

## 1. Introduction

The gastrointestinal tract, the primary organ responsible for digestion, absorption, secretion, and immune function in ruminants like cows, harbors a rich and diverse microbial population. The microbial community, which coexists with the host and undergoes changes based on the host’s physiological state, plays a vital role in various aspects of the host’s nutrition, metabolism, and immune function (1). Despite the relatively small volume of the gastrointestinal microbiota, its total number exceeds 100 trillion, exerting a significant influence on the overall health and physiology of the host (2). The composition and structure of the gut microbial are crucial for maintaining the stability and functionality of the host’s gastrointestinal system. Microbial fermentation in the cow’s gastrointestinal tract serves as the foundation for ruminant production and is a key factor enabling cows to convert low-quality feed into high-quality milk and meat. This intricate ecosystem consists of diverse microorganisms, including bacteria, protozoa, and fungi.

Currently, bacteria are the most extensively studied within the cow gut microbiota. These bacteria play a vital role in the digestion of plant materials by producing enzymes that break down fiber into volatile fatty acids (VFAs), proteins, vitamins, and other nutrients. These metabolites contribute to the nutritional requirements of cows and the influences metabolic processes that ultimately affect productivity traits (3, 4). Moreover, these bacteria are involved in energy conversion, nutrient absorption and even participating in inflammation and tissue deposition processes that impact the overall life activities of cows (5). Recent studies have revealed that changes in the quantity and abundance of cow gut microbiota occur with age and variation in feed intake. These changes play a crucial role in guiding nutrient digestion, absorption and immunity response within the cow’s body (6–9).

For instance, the *Firmicutes*/*Bacteroidetes* ratio in the cow’s gastrointestinal tract (GIT) shows a positive correlation with milk fat yield. Variations in the abundance change of GIT microbes are associated with genes related to milk fat production, indicating a close relationship between dairy performance traits and GIT microbes (10). Additionally, significant correlations have been observed between the expression levels of lipid-metabolism-related genes ad intestinal mucosal immune genes with GIT microbes, highlighting the essential role of these microbes as “secretory organs” that contribute to internal energy balance and immune regulation (11). The community structure of the cow gut microbiota is the result of co-evolution between prokaryotes and eukaryotes. Understanding the physiological characteristics and functions of cow gut microbiota is crucial for comprehending its pivotal role in production and health, making it a hot topic in research fields such as dairy breeding, nutrition, and reproduction. However, the regulatory mechanisms governing the cow gut microbiota are still not fully understood. This review aims to summarize the characteristics and physiological effects of cow gut microbiota, providing a theoretical foundation for further studies on the utilization of gastrointestinal microorganisms in dairy cattle breeding and productivity improvement. It also serves as a valuable reference for research on gastrointestinal microbial communities in other ruminants.

## 2. Structure of the gastrointestinal tract of cow

A cow’s gastrointestinal tract consists of several parts, including the stomach, duodenum, jejunum, ileum, cecum, and rectum. The stomach of a cow has four chambers: rumen, reticulum, omasum, and abomasum (also known as the true stomach). The rumen is the largest chamber, comprising about 80% of the total stomach volume, and it possesses a large capacity and a complex microbial ecosystem (12). The small intestine in cows is composed of the duodenum, jejunum, and ileum, with an average length of 7–8 m in adult cows. On the other hand, the hindgut consists of the cecum, colon, and rectum, with an average length of 5–5.6 m in adult cows (13). The digestion process in cows begins in the rumen, followed by the reticulum, omasum, and abomasum. In the rumen, microbial fermentation and mechanical action help break down crude fiber into easily absorbable nutrients. The reticulum and omasum aid in further digestion and absorption. The abomasum, considered the true stomach, mainly digests protein from the partially digested food (chyme). As chyme enters the duodenum, it undergoes additional breakdown by digestive enzymes released from the pancreas, as well as the action of bile juice. The small intestine, particularly the duodenum, jejunum, and ileum, is responsible for nutrient absorption, facilitated by the presence of villi that increase the surface area of the intestinal wall, enhancing nutrient absorption capacity. Any remaining food residues move into the hindgut, specifically the cecum, where further microbial fermentation takes place. The final product, feces, is then excreted through the rectum (14, 15).

## 3. Composition and characteristics of microbes in gastrointestinal tract of cows

Microorganisms are ubiquitous in the environments where animals reside, as long as suitable nutritional conditions are present for their growth. Surfaces exposed to the external environment, such as the skin, oral cavity, gastrointestinal tract, respiratory system, and reproductive system, all harbor microbial communities. Among these, the gastrointestinal tract provides a particularly favorable environment for microbial colonization due to the availability of nutrients. The number of microbes present in the gastrointestinal tract is remarkably high, exhibiting extensive genetic diversity.

Studies have revealed that the gastrointestinal microbiota of cows comprises over 1,800 genera and 40,000 species of bacteria, with the total number of bacteria reaching billions. These numbers are 10–100 times higher than the numbers of host cell. Additionally, the plasmid gene numbers carried by these microbes is approximately 150 times higher than the number of genes found in hosts (1, 2, 10). Microbes colonize various regions throughout the entire structure of the cow’s gastrointestinal tract, working synergistically to degrade fiber in the feed. They produce volatile fatty acids, proteins, vitamins, and other substances that meet the nutritional requirements of the host nutritional.

The rumen of an adult cow can have a volume of up to 180 L and contains approximately 10^10^~10^11^ bacteria/mL, 10^6^ ~ 10^8^ archaea/mL, 10^4^ ~ 10^6^ protozoa/mL and 10^3^ ~ 10^6^ fungi/mL (3, 16). Among these microorganisms, bacteria are the most abundant in rumen and can be categorized into four subgroups: liquid-phase bacteria, solid-phase bacteria, rumen epithelial adherent bacteria, and eukaryotic-associated bacteria. Studies have demonstrated that the rumen exhibits the highest diversity of bacterial species compared to other parts of the digestive system, with a total of 47 genera, including 16 specific ruminal genera. The dominant phyla in the rumen are *Firmicutes*, *Bacteroidetes*, and *Prevotella*, *Fibrobacter*, and *Succinivibrio* are major genus (17, 18).

As the digestive process progresses from the rumen to the small intestine and then the hindgut, the acidity level gradually decreases, accompanied by a reduction in oxygen content, resulting in an increase in both the quantity and abundance of gut microbiota. In the small intestine and hindgut, bacteria are the predominant microorganisms in the cow’s gut microbiota. The small intestine contains approximately 10^9^–10^10^ bacteria/mL, with *Firmicutes* and *Proteobacteria* being the dominant phyla. In the hindgut, there can be approximately 10^12^ ~ 10^14^ bacteria/mL, with *Firmicutes*, *Bacteroidetes*, *Actinobacteria*, and *Verrucomicrobia* as the dominant phyla. Furthermore, the relative abundance of these bacterial phyla in the small intestine and hindgut is higher than that in the rumen (14, 19) (as shown in Table 1).

**Table 1:** Distribution and magnitudes of gastrointestinal microbes in dairy cows.

	Dominant bacteria	Abundance	Regulation	References
Rumen	*Firmicutes*, *Bacteroidetes*, and *Proteobacteria* phyla	10^10^–10^11^ cells/mL	Providing volatile fatty acids, microbial proteins, vitamins to the host by fermentation to meet 70% of the host’s energy needs.	(3, 16–18)
Small intestine	*Firmicutes* and *Proteobacteria* phyla	10^9^–10^10^ cells/mL	Further breaking down chyme and absorbing nutrients	(14, 19)
Hindgut	*Firmicutes*, *Bacteroidetes*, *Actinobacteria*, and *Verrucomicrobia* phyla	10^12^–10^14^ cells/mL	Fermenting and decomposing fibrous xylose and hemicellulose to meet 30% of the host’s energy needs, and participating immunity.	(14, 19)

Numerous studies have highlighted distinct differences in the microbial communities found in the rumen, small intestine and hindgut of dairy cows (5, 10, 20). These differences have been shown to have a direct impact on immune function and production traits in cows (21, 22). Therefore, the composition of cow’s gut microbiota plays a crucial role in determining their health status and production traits. Investigating the patterns of variation and exploring the interactions between the cow’s gut microbiota and the hosts has become a prominent and active area of research in the fields of cattle breeding and nutrition.

## 4. Physiological functions of cow gastrointestinal microorganisms

As research on gastrointestinal microorganisms in animals progresses, there has been a shift in focus from changes in composition, abundance, and diversity to understanding their biological functions. Studies have revealed that the gastrointestinal microorganisms in cows possess a wide range of metabolic capabilities and diverse biological functions. They maintain a symbiotic relationship with the host’s gastrointestinal mucosa and rely on the dietary components and shed epithelial cells as sources of nutrition (5, 23). These microorganisms primarily perform metabolic functions such as the uptake and conversion of fatty acids and amino acids. They also play a protective and defensive rile by reducing the absorption of toxic substances and preventing the invasion of harmful bacteria or pathogens. Furthermore, they have a structural function in regulating host immunity through interactions with gastrointestinal mucosa, acting as an intestinal barrier (1, 16).

### 4.1. Nutritional and metabolic functions of rumen microbiota in dairy cows

Dairy cows and other ruminants possess an impressive ability to convert low-quality fibrous feed into high-quality milk and meat products. This remarkable feat is made possible, in large part, by the rumen microbiota (as shown in Table 2).

**Table 2:** The functions of cow gastrointestinal microorganisms in production and health.

Microorganisms	Classification	Beneficial function	Mechanism	References
*Prevotella*	*Bacteroidetes*	Metabolic regulation	Digesting cellulose and produce volatile fatty acids to facilitate the milk fat synthesis; Metabolically affecting the concentrations of glutathione, phenylalanine and galactose in rumen, and then affect the concentrations of glycine, serine and threonine in serum of dairy cows, thereby regulating the production of milk fat and milk proteins	(24–27)
*Succinimonas amilolytica*	*Pseudomonadota*			
*Clostridium aerogenes*	*Firmicutes*	Metabolic regulation	Fermentation cellulose to maintain normal gas pressure in the rumen to facilitate the digestion and emptying of food; regulating the host’s food intake behavior	(28–33)
*Actinomyces*	*Actinomycetota*			
*Methanobrevibacter*	*Euryarchaeota*	Metabolic regulation	Digesting cellulose in the rumen produces large amounts of greenhouse gasses such as methane	(28, 34, 35)
*Acetobacter*	*Pseudomonadota*	Metabolic regulation	Producing alpha-ketoglutaric acid, which ultimately promotes milk fat production in cows	(36)
*Saccharofermentan*	*Firmicutes*			
*Enterococcus faecalis*	*Firmicutes*		Effectively reduce the activity of Methanogenium by producing myristic acid, and ultimately increase milk fat production	(37, 38)
*Bacteroidetes*	*Bacteroidota*	Metabolic regulation and immune regulation	Regulation of gluconeogenesis to facilitate the milk fat synthesis	(39, 40)
*Bacteroides*			Conjugated linoleic acid can be synthesized to participate in lipid metabolism and immune regulation; inducing the differentiation of T_reg_ cells	(41, 42)
*Bacteroides intestinalis*			The primary bile acid can be conjugated to the secondary bile acid to facilitate the milk fat synthesis	(41)
*Proteobacteria*	*Proteobacteria*	Metabolic regulation	Producing choline and involving in phospholipid formation and unsaturated fatty acid transport in the host	(43, 44)
*Atopobium*	*Actinomycetota*	Metabolic regulation and immune regulation	Causing rumen acidosis; causing mastitis through breakdown of the blood-milk barrier	(45–50)
*Desulfocurvus*	*Pseudomonadota*			
*Lactobacillus*	*Firmicutes*			
*Ruminococcus*				
*Clostridium*				
Segmented filamentous bacteria	*/*	Immune regulation	Inducing Th17 cells	(51, 52)
*Bifidobacterium*	*Actinomycetota*	Immune regulation	Regulating mastitis through the gut-stomach axis and the gut-blood axis	(53)
*Peptostreptococcaceae*	*Firmicutes*	Immune regulation		
*Enterococcu*	*Enterococcu*	Immune regulation	Inducing mastitis	(54)
*Sterptococcus*	*Firmicutes*	Immune regulation		
*Lactobacillus rhamnosus*	*Firmicutes*	Immune regulation	Reducing the risk of oxalate stones forming in the kidneys through the gut-kidney axis	(55, 56)
*Bifidobacterium*	*Actinomycetota*	Metabolic regulation and immune regulation	Improving blood sugar control, lower lipid levels through the gut-stomach axis, and reducing the risk of oxalate stones forming in the kidneys gut- kidney axis	(56–58)
*Bacteroides thetaiotaomicron*	*Bacteroidota*	Immune regulation	Promoting the repair of damaged epithelial cells and maintain the desmosomal structure of intestinal villi	(59, 60)
*Lactobacillus rhamnosus GG*	*Firmicutes*	Immune regulation	Preventing the apoptosis of intestinal epithelial cells induced by cytokines	(61)

#### 4.1.1. Rumen microbiota

The fermentation of microorganisms in the rumen is the foundation of dairy cow production. These microorganisms maintain a synergistic and symbiotic relationship, collectively providing the majority of enzymes required to break down feed substrates. Through fermentation, they degrade cellulose, protein, and lipids in plant feed, producing volatile fatty acids (VFAs), microbial proteins, and vitamins as nutrients for the host. These nutrients serve as substrates for energy metabolism, meet 70% of the host’s energy needs for physiological activities (62). Studies have shown that in cows with higher feed conversion rates, the diversity and abundance of rumen microorganisms are lower, but there are more fiber-degrading bacteria. On the other hand, cows with lower feed conversion rates exhibit higher diversity and abundance of microorganisms, particularly those that produce gas (28, 29). For instance, *Prevotella* and *Succinimonas amilolytica* are relatively abundant in cows with higher feed conversion rates. These microorganisms exhibit strong cellulose digestion functions in carbohydrate-digesting enzyme activities, leading to higher concentrations of VFAs. Short-chain fatty acids enter the small intestine where they are emulsified by bile and synthesized into triglycerides in the intestinal mucosal cells. They are then transported to the bloodstream in the form of chylomicrons, very-low-density lipoproteins, low-density lipoproteins, and high-density lipoproteins. After enzymatic breakdown through capillary walls, they become free fatty acids and glycerol that are taken up by the mammary gland (25, 26). Within the mammary epithelial cells, a series of enzymes acts on them to re-synthesize triglycerides by combining with glycerol. They are then incorporated into the cytoplasm as lipid droplets, combined with lipoproteins such as phospholipids, and form milk fat globules. These globules are secreted extracellularly through apocrine secretion and enter the milk, contributing to the formation of milk fat (27). Additionally, these microorganisms can potentially regulate the expression of key genes in rumen epithelial cells, enhancing cellulose digestion and the absorption and metabolism of nutrients such as VFAs. This ultimately influences fat metabolism and the synthesis of milk components such as milk fat and milk protein in mammary epithelial cells (27). In cows with lower feed conversion rates, microorganisms such as *Clostridium aerogenes*, *Actinomyces*, and *Methanobrevibacter* have higher abundance in the rumen. These microorganisms utilize cellulose fermentation to produce gas, maintaining normal gas pressure in the rumen to promote food digestion and emptying. However, *Methanobrevibacter*, which is more abundant in this context, produces a significant amount of methane and other greenhouse gasses through cellulose digestion in the rumen. This not only reduces feed conversion rates but also has a negative impact on global climate change (28, 34, 35). These findings demonstrate that rumen microorganisms can affect the feed conversion rate and, consequently, the production traits of beef or dairy cattle through their nutritional metabolism functions.

Furthermore, rumen microorganisms can produce a large number of small-molecule metabolites, which play a crucial role in microbial information transmission and directly participate in the host’s nutritional metabolism, transcriptional mechanisms, and epigenetics, enabling communication between the microbiota and host cells (63). Studies have found that rumen microorganisms can synthesize fatty acids, particularly polyunsaturated fatty acids. These polyunsaturated fatty acids enter the small intestine in the form of phospholipids, forming microbial phospholipids. By increasing the proportion of microbial phospholipids in the small intestine, the content of unsaturated fatty acids in milk can be significantly improved, thereby addressing the lower unsaturated fatty acid content in some milk samples (64). *Prevotella* and *Succinimonas amilolytica* in the rumen can influence the concentrations of substances such as glutathione, phenylalanine, and lactose, thereby affecting the concentrations of metabolites such as glycine, serine, and threonine in cow serum, ultimately regulating milk fat synthesis (24). Certain genera of short-chain bacteria in the rumen can produce methionine, which promotes *Acetobacter* and *Saccharofermentans* to produce more alpha-ketoglutarate, ultimately facilitating milk fat synthesis (36). *Enterococcus faecalis* can effectively reduce the activity of *Methanogenium* in the rumen by producing cinnamic acid, resulting in decreased energy conversion to methane gas, improved feed conversion efficiency, and increased milk fat production (38). Similar findings have been observed in other ruminant animals, where rumen bacteria can regulate gluconeogenesis by modulating bile acid metabolism. They can also promote cholesterol excretion by metabolizing bile salts, thereby regulating the concentration of triglycerides in the blood (39, 40). Consequently, an increasing number of studies are attempting to identify specific microorganisms or their metabolites in the rumen of dairy cows to significantly improve production traits. Additionally, feeding specific diets and additives to enhance the abundance of beneficial microorganisms in the rumen is also being explored.

#### 4.1.2. Intestine microorganisms

The microbial community in the cow’s intestinal tract, as a relatively independent component, primarily participates in host nutrient metabolism through heat production and metabolic functions. The digestion of food in the cow’s rumen forms chyme, which has a high water content and low mass transfer resistance. Due to the fast peristalsis frequency in the small intestine, chyme stays for a short time in the small intestine. However, when it reaches the large intestine, the emptying of food residue occurs at a slower rate compared to the small intestine, as the cross-sectional area of the large intestine is approximately four times larger. This provides enough time for fermentation and decomposition of the nutrients remaining in the chyme by the microbial community in the hindgut. Although chyme is primarily fermented and digested by the microbes in the rumen and small intestine, certain components such as indigestible cellulose require the action of hindgut microbes for digestion. Studies have shown that approximately 30% of fibrous, xylose and hemicellulose are fermented and decomposed in the hindgut, indicating that the fermentation processes in hindgut remain an essential energy source for cows (65). Studies have also found that the microbial community in the colon content or feces of ruminants can utilize the undigested food residues in the intestinal tract to produce approximately half of the energy needed by the host (30). Moreover, the number of metabolic enzymes produced by intestinal microbes is much greater than those produced by host tissues such as the liver. For example, bacteria from the phyla *Bacteroidetes*, *Firmicutes*, *Proteobacteria*, and *Actinobacteria*, can decompose semi-cellulose, which cannot be digested by the cow’s rumen, and produce short-chain fatty acids that are absorbed by the intestinal mucosa and serve as substrates for energy metabolism. These short-chain fatty acids are then transported through the blood to various organs, providing energy (30). Additionally, these short-chain fatty acids can stimulate intestinal peptide secretion and participate in the regulation of energy metabolism (66). *Bacteroidetes* bacteria contain various glycosyltransferases, glycoside hydrolases, and polysaccharide lyases, which are essential enzymes for carbohydrate metabolism in cows (67). Furthermore, *Bacteroidetes* bacteria can synthesize conjugated linoleic acid, which participates in lipid metabolism and immune regulation. *Bacteroides intestinalis* can convert primary bile acids into secondary bile acids as they are reabsorbed and enter lipid metabolism pathways through chylomicrons (41). The proteases expressed by intestinal microbes can also work together with host proteases to regulate protein metabolism and conversion (68).

In addition, the intestinal microbiota can participate in host nutrient metabolism by producing a large number of small-molecule metabolites. For example, bacteria from the phyla *Bacteroidetes*, *Firmicutes*, *Proteobacteria*, and *Actinobacteria* produce short-chain fatty acids, lipopolysaccharides, and peptidoglycans, which not only directly participate in host metabolism but also regulate food intake behavior through the gut-brain-adrenal axis, producing pancreatic glucagon, growth hormone, and ghrelin (30–33). *Firmicutes* bacteria can produce carnitine, which activates the formation of brown adipose tissue and beige adipose tissue in the host through the circulation, participating in lipid metabolism (37). *Actinobacteria* bacteria can produce choline, which participates in phospholipid formation and the transport of unsaturated fatty acids in the host (43). Relevant studies have also found that high levels of choline can increase the sensitivity of milk fat synthesis in cows (44). These nutritional metabolism functions of the gut microbiota play a dominant role, particularly in the developing rumen of young calves, highlighting the importance of the nutritional metabolism function of the cow’s intestinal microbiota alongside the rumen microbiota.

### 4.2. Immune regulatory function of the gastrointestinal microbiota in dairy cows

The mucosa of the cow’s gastrointestinal tract consists of the epithelial layer, lamina propria, and muscular layer. The gastrointestinal immune system includes gastrointestinal-associated lymphoid tissue, effector T cells, regulatory T cells, IgA-producing B cells, and intrinsic lymphocytes in the lamina propria. Among them, the lamina propria contains more immune cells such as B cells, T cells, plasma cells, and monocytes/macrophages than any other tissue, enabling them to respond to the invasion of billions of bacteria by the body (69). Particularly, the intestinal microbiota in the hindgut is directly related to the establishment and immune homeostasis of the host’s immune system, making the gastrointestinal tract one of the largest peripheral immune organs in the body and an important part of the overall immune system (70). The microbial community in the gastrointestinal tract, while participating in host nutrient metabolism, also plays a protective and defensive role by regulating host immunity and maintaining the intestinal barrier. For example, the microbiota in the cow’s rumen can produce volatile fatty acids through fermentation for host energy metabolism. However, feeding high-concentrate diets can increase the relative abundance of certain bacteria such as *Atopobium*, *Desulfocurvus*, and *Lactobacillus*, while also increasing the presence of toxic compounds such as endotoxins, histamine, and ethyl acetate, leading to rumen acidosis. Furthermore, lactobacilli, rumenococci (Ruminococcus), and clostridia (Clostridium) in the rumen can express bile salt hydrolase and produce substances such as acetate salts and lipopolysaccharides. These substances can disrupt the blood-milk barrier and lead to mastitis, affecting the production traits of ruminant animals (45–47).

The intestinal microbiota, as the largest and most complex microbial ecosystem in the host’s body, plays a crucial role in bridging the gap between diet and the host. It can regulate both the innate and adaptive immune responses of the host’s intestinal immune system. For example, compared to germ-free mice, specific pathogen-free (SPF) mice have more mature lymphoid tissues, as well as increased numbers of Th17 cells in the small intestine, regulatory T cells (Treg) in the colon, and intraepithelial lymphocytes (IELs) with αβ T cell receptors. These immune cells contribute to immune tolerance (51). Transplantation of segmented filamentous bacteria (SFB) in mouse intestines can effectively induce activation of Th17 cells (52), while transplantation of *Clostridium* or *Bacteroides* can further induce differentiation of Treg cells in the colon (42). The intestinal microbiota can also promote the differentiation of B cells into plasma cells, leading to increased secretion of IgA. It stimulates intestinal epithelial cells to secrete antimicrobial peptides and induces goblet cells to release mucus, thus defending against the invasion of exogenous pathogenic microorganisms (71). Additionally, various innate lymphoid cells in the intestines can be stimulated by the microbiota and its metabolites to produce interleukins. For example, short-chain fatty acids can induce the production of IL-18 by intestinal epithelial cells, promote tolerogenic dendritic cells to express IL-10, and further enhance the release of antimicrobial peptides by intestinal epithelial cells, thereby maintaining the balance of intestinal microbial communities and protecting against the invasion of exogenous pathogens (72, 73). After the “gut-mammary” hypothesis was proposed by Arroyo et al., suggesting that microorganisms in the intestines could enter the mesenteric lymph nodes and be transferred to the mammary glands (74), an increasing amount of research has demonstrated the impact of the gut microbiota on various aspects of host health, metabolism, and diseases through axes such as the gut-brain axis, gut-stomach axis, gut-liver axis, and gut-pancreas axis (75–77). For example, Young et al. found a small number of operational taxonomic units (OTUs) belonging to rumen bacteria, *Bifidobacterium*, and *Peptostreptococcaceae* in the feces, milk cells, and blood leukocytes of cows (53). Ma et al. discovered that both the fecal microbiota and milk microbiota of cows with mastitis showed higher levels of *Enterococcus*, *Streptococcus*, and *Staphylococcus* and lower levels of *Lactobacillus* (54). When fecal microbiota from cows with mastitis was transplanted into germ-free mice, the mice also developed mastitis (48). On the other hand, acetates and butyrates produced by *Firmicutes* can reduce the severity of *Staphylococcus aureus*-induced mastitis by altering the blood-milk barrier (49). *Lactobacillus rhamnosus* can alleviate NLRP3 inflammasome activation induced by *Escherichia coli* and inhibit oxidative cluster-mediated cell apoptosis and inflammatory responses (55). Furthermore, Hu et al. found that the short-chain fatty acids produced by *Firmicutes* and *Bacteroides* in the cow’s gut can prevent lipopolysaccharides produced in the rumen from entering the bloodstream and mammary tissue, thus protecting mammary gland lactation function (50). *Bifidobacterium* can improve digestion, enhance blood glucose control, and lower lipid levels through the gut-stomach axis (57, 58). Factors secreted by intestinal microbiota, such as GLP-1, ghrelin, and 5-HT, can regulate the metabolic balance of blood glucose in the host by influencing the α/β cell function of the pancreas, supporting the gut-pancreas axis theory (78). Intestinal bacteria such as *Lactobacillus* and *Bifidobacterium* can reduce the production of oxalate during carbohydrate fermentation and bacterial metabolism, thus lowering the risk of kidney oxalate stone formation, demonstrating the regulation of the gut-kidney axis (56). Additionally, the intestinal microbiota can exert immune effects by participating in the metabolism of other substances. For example, they can remove the glycosylated portion of polyphenols to produce active metabolites, which enter the bloodstream and reach distant tissues or organs to exert immunomodulatory effects (79).

### 4.3. Defense function of gastrointestinal microorganisms in cows

The gastrointestinal microbiota in dairy cows plays an important role in maintaining the integrity of the gastrointestinal structure and function, as well as the mucosal structure. Particularly, a large amount of mucus adheres to the gastrointestinal mucosa, which helps balance the relationship between the microbiota and the host. For example, an overabundance of Gram-negative bacteria in the rumen can lead to abnormal fermentation and excessive production of lactate. Additionally, the outer membrane of Gram-negative bacteria primarily consists of lipopolysaccharides (LPS). The accumulation of lactate and LPS can damage rumen epithelial cells, causing rumen acidosis, and also allow harmful substances to enter the bloodstream, leading to mastitis in dairy cows (11). Furthermore, a study by Zhong et al. comparing the rumen and hindgut microbiota of dairy cows under different udder health conditions found that the hindgut microbiota and metabolites were more closely associated with udder health than the rumen microbiota (21).

Research has shown that microbes in the hindgut can promote the secretion of mucin, increase the thickness of the mucus layer, and provide more adhesive sites for beneficial bacteria, thereby strengthening the barrier function that inhibits the invasion of various pathogenic bacteria. For instance, hindgut microbes can regulate the transcription of angiogenin-3 (Ang-3) gene to promote the development of intestinal mucosal structure (80). *Bacteroides thetaiotaomicron* can not only promote the expression of fucose on the surface of intestinal epithelial cells (59), which facilitates the repair of damaged epithelial cells, but also induce the expression of small proline-rich protein 2A (SPRR2A), rich in proline, in intestinal epithelial cells to maintain the structure of intestinal epithelial microvilli and desmosomes (60). *Lactobacillus rhamnosus GG* can produce soluble proteins that effectively inhibit cytokine-induced apoptosis of intestinal epithelial cells (61).

In addition, various amino acid transporters present on the cell wall of gastrointestinal bacteria can transport amino acids from the intestinal lumen into bacterial cells, where they are converted into signaling molecules and antimicrobial peptides to participate in the host’s gastrointestinal defense processes. For example, the histidine decarboxylase encoded by the hdcA gene in bacteria can convert L-histidine into histamine, which effectively inhibits the invasion of various pathogenic bacteria (81). Moreover, the enzymes expressed by the gut microbiota can not only metabolize carbohydrates, amino acids, and lipids, but also break down toxic substances. For instance, the gut microbiota contains abundant soluble cytochrome P450 (CYP450), which plays a defensive role by metabolizing xenobiotics (82).

## 5. Feeding practices to modulate gastrointestinal microbiota and improve dairy cow production and health

Ruminant animals, including cows, primarily rely on fiber as their main source of feed. Therefore, providing cows with fiber-rich feed such as forage and high-fiber hay can supply the substrates and energy required by beneficial gastrointestinal bacteria, promoting the growth and reproduction of beneficial microorganisms. Feeding silage and acidic fermented feed, such as corn silage, acetic acid-treated corn, and fermented alfalfa, creates an acidic environment necessary for the growth of lactic acid bacteria and other beneficial microorganisms. However, it is important to note that high-fiber feed can also stimulate the production of methane-producing bacteria, resulting in decreased feed efficiency. Conversely, a diet higher in concentrate or acidity can lower the pH in the cow’s gastrointestinal tract, potentially causing subacute ruminal acidosis (SARA) (83).

Therefore, an increasing number of studies have attempted to improve the growth and metabolic activity of beneficial bacteria in the gastrointestinal tract by adding probiotics such as *Lactobacillus* and Saccharomyces to the cow’s feed or drinking water. This promotes the interaction between probiotics and the gut microbiota, stimulating the growth and metabolic activity of beneficial bacteria (84, 85). Additionally, feed components rich in prebiotic fibers, such as inulin (86), fructo-oligosaccharides (87), and xylo-oligosaccharides (88), provide the necessary energy and substrates for beneficial microorganisms. Extracts from dandelion (89), honeysuckle (90), seaweed (91), and others have been found to possess antibacterial and anti-inflammatory properties, exerting a positive influence on the gut microbiota. Furthermore, the addition of inhibitors such as 3-nitrooxypropanol (3-NOP) to the diet can suppress the production of greenhouse gasses, including methane, by inhibiting methyl-coenzyme M reductase (MCR) and improving feed efficiency (92). Another approach involves removing protozoa that coexist with methanogens, thereby disrupting interspecies hydrogen transfer and reducing the production of methane gas (86, 93). These measures not only do not affect milk production but can also enhance milk fat content (94). These observed results provide direct evidence for programming the rumen microbiota of cows through dietary interventions or gene selection.

## 6. Conclusion

With the continuous advancements in biotechnology and omics technologies, various factors regulating production traits of livestock and poultry have been widely studied (95–98), and the efficient modulation of gastrointestinal microbiota in dairy cows for production traits and immune health is unquestionable. This review summarizes the relationship between dairy cow gastrointestinal microbiota and host production traits and health. It is known that the quantity of gastrointestinal microbiota in dairy cows surpasses that of host cells and genes by several orders of magnitude. These microorganisms can metabolize various substances such as cellulose and polysaccharides, providing abundant enzymes to enhance the metabolic function of dairy cows. They also produce a variety of metabolites, including vitamins, to meet the nutritional and immune needs of cows, enabling them to convert low-quality feed into high-quality meat and dairy products. Furthermore, these microorganisms provide a balanced and coordinated buffering space in the complex gastrointestinal environment. This balance not only maintains the abundance and diversity of beneficial bacteria within appropriate ranges but also provides defensive functions such as decomposition of harmful substances and maintenance of gastrointestinal structure, preventing the overgrowth and invasion of pathogenic bacteria. Therefore, studying the dynamics of gastrointestinal microbiota and its interdependence with the host has become a hot topic in the fields of dairy cattle breeding, nutrition, and reproduction.

Although significant progress has been made in understanding the gastrointestinal microbiota of livestock and poultry, including dairy cows, in the past two decades (99–105), there is still a lack of evidence demonstrating strong causal relationships between gut microbiota and host production traits, especially in terms of the functionality of hindgut microbiota in ruminants, which is still in its early stages of research. Currently, studies on microbiota, whether based on the rumen or hindgut, often rely on correlation analyses after grouping based on differences in production performance or immune indicators. However, there is often a lack of clear biological relevance, making it difficult to establish causal relationships between these microorganisms and the differential traits. Additionally, despite our comprehensive search strategy, it is possible that some important studies, particularly those published after our review, may not have been included, resulting in limitations in our review. Therefore, in future in-depth research, apart from avoiding our limitations, it is necessary to shift the focus of studying the association between microorganisms and host traits toward investigating causal relationships and mechanisms, ultimately identifying microorganisms and microbial pathways that can positively regulate host production traits and immune performance.

## Author contributions

LL and QZ: conceptualization. LL, PW, AG, YY, FC, and QZ: investigation and supervision. LL: writing—original draft preparation, project administration, and funding acquisition. LL and PW: writing—review and editing. All authors have read and agreed to the published version of the manuscript.

## Funding

This work was supported by the National Natural Science Foundation of China (Grant No. 31902152), Yunnan Province’s “Xingdian Talent” Youth Talent Project, Southwest Forestry University Scientific Research Fund (Grant No. 112119), and Yunnan Provincial Department of Education Scientific Research Project Fund (Grant No. 2018JS333).

## Conflict of interest

The authors declare that the research was conducted in the absence of any commercial or financial relationships that could be construed as a potential conflict of interest.

## Publisher’s note

All claims expressed in this article are solely those of the authors and do not necessarily represent those of their affiliated organizations, or those of the publisher, the editors and the reviewers. Any product that may be evaluated in this article, or claim that may be made by its manufacturer, is not guaranteed or endorsed by the publisher.
